# Diseases of the Eye, Ear, Throat and Nose

**Published:** 1893-08

**Authors:** 


DISEASES OF THE EYE, EAR, THROAT AND NOSE, by Frank E. Miller, M. D., Throat Surgeon, Vanderbilt Clinic College of Physicians and Surgeons, New York, James P. McEvoy, M. D., Throat Surgeon, Bellevue Hospital, Out-Patient Department, New York, and John E. Weeks, M. D., Lecturer on Ophthalmology and Otology, Bellevue Hospital Medical College, New York. Being Volume 10 of the Students' Quiz Series, edited by Bern B. Gallaudet, M. D., Demonstrator of Anatomy, College of Physicians and Surgeons, New York; Visiting Surgeon, Bellevue Hospital, New York. Pocket-size 12mo., 218 pages, with 89 illus. and 2 full-page plates. Limp cloth, $1.00. From Lea Brothers & Co., Philadelphia.

Uniform with the other volumes of the Students Quiz Series, this little volume is one of the best that has come to our hands. Concise, plain and brought fully up to date, it will no doubt prove of great value to students and practitioners who wish an epitome of the anatomy, diagnosis, treatment, etc., and diseases of the parts that it comprises. The illustrations and mechanical work are unsurpassed. G. B.

